# Reply: The importance of disrupting complement activation in acute lung injury

**DOI:** 10.1172/JCI182451

**Published:** 2024-06-17

**Authors:** Simon J. Cleary, Mark R. Looney

**Affiliations:** 1 Department of Medicine, UCSF, San Francisco, USA.; 2 Institute of Pharmaceutical Sciences, King’s College London, London, United Kingdom.

**Keywords:** Immunology, Pulmonology, Complement

**The authors reply:** We appreciate the letter from Kapur and colleagues and are glad to be in agreement on the importance of the complement cascade in experimental transfusion-related acute lung injury (TRALI) (1). We have also measured increased complement activation in clinical TRALI (2), but as complement deposition and lung injury occur within a few minutes in mouse models of TRALI (2, 3), and various mitigation strategies have been effective in preventing clinical TRALI (4), we struggle to take an optimistic view on therapeutic or prophylactic use of complement inhibitors to ameliorate or prevent TRALI. We are hopeful that recombinant IgG Fc hexamers or complement therapeutics might improve outcomes in patients at high risk of developing antibody-mediated rejection (AbMR) following solid-organ transplantation or in other diseases, such as COVID-19. Recent approvals of therapeutics targeting IgG (imlifidase for desensitization to prevent AbMR, ref. 5) or C1s (sutimlimab for cold agglutinin disease, ref. 6) demonstrate that inhibiting “upstream” antibody effectors is feasible, and these approaches would be expected to preserve some of the immune protection mediated by membrane attack complexes. Determining the importance of IgG Fc-Fc interactions and different complement components in antibody-mediated diseases will help to improve diagnostics and therapeutics.

## Version 1 06/17/2024

Electronic publication
